# Title Effects of Antioxidant Treatments on Sprouting, Survival, Callus Formation, and Attempted Adventitious Rooting of *Campomanesia adamantium* Cuttings Across Different Seasons

**DOI:** 10.3390/plants15131990

**Published:** 2026-06-27

**Authors:** Gustavo Henrique Oliveira Lira Chaves, Luiz Henrique Rodrigues Guimarães, Romário Ferreira Cesário, Francielly Rodrigues Gomes, Maria Eduarda Souza Moraes, Pablo Moura Simão, Isabelly da Silva Gonçalves, Joanda Ferreira Alexandre da Silva, Givanildo Zildo da Silva, Mayara Cristina Lopes, Ricardo Fagundes Marques, Aracy Camilla Tardin Pinheiro Bezerra, Piero Iori, Luciana Celeste Carneiro, Hildeu Ferreira da Assunção, Simério Carlos Silva Cruz, Vinícius Coelho Kuster, Alejandro Hurtado Salazar, Danielle Fabíola Pereira da Silva

**Affiliations:** 1 Institute of Agricultural Sciences, Federal University of Jataí, Jataí 75801-615, Goiás, Brazil; gustavo.henriquelira@discente.ufj.edu.br (G.H.O.L.C.); romarioengagricola@gmail.com (R.F.C.); fram_rodgomes@hotmail.com (F.R.G.); moraes.maria@discente.ufj.edu.br (M.E.S.M.); pablo.simao@discente.ufj.edu.br (P.M.S.); isabellygoncalves@discente.ufj.edu.br (I.d.S.G.); joanda.silva@discente.ufj.edu.br (J.F.A.d.S.); rfmarques94@gmail.com (R.F.M.); aracy.bezerra@ufj.edu.br (A.C.T.P.B.); pieroiori@ufj.edu.br (P.I.); luciana_celeste_carneiro@ufj.edu.br (L.C.C.); hildeu@ufj.edu.br (H.F.d.A.); simerio@ufj.edu.br (S.C.S.C.); daniellefpsilva@ufj.edu.br (D.F.P.d.S.); 2 Institute of Agricultural Sciences, University of Rio Verde, Rio Verde 75901-970, Goiás, Brazil; givanildo@unirv.edu.br (G.Z.d.S.); mayara@unirv.edu.br (M.C.L.); 3 Institute of Biosciences, Federal University of São João del-Rei, Sete Lagoas 35701-910, Minas Gerais, Brazil; viniciuskuster@ufsj.edu.br; 4 Producción Agropecuaria, University of Caldas, Manizales 170004, Caldas, Colombia; alhuza@gmail.com

**Keywords:** rhizogenesis, phenolic compounds, plant propagation, cerrado species, vegetative propagation

## Abstract

Gabiroba (*Campomanesia adamantium* (Cambess.) O. Berg) is a native fruit tree of the Brazilian Cerrado biome with notable economic potential and phytotherapeutic properties. However, commercial seedling production is limited by the recalcitrant nature of its seeds and vegetative propagation is further constrained by phenolic compound oxidation. Therefore, this study evaluated the effects of antioxidant treatments on sprouting, cutting survival, callus formation and attempted adventitious rooting of *C. adamantium* cuttings collected in different seasons. The experiment was conducted in a greenhouse at the Federal University of Jataí using cuttings collected from an experimental orchard. Eleven treatments were evaluated: a control and different concentrations of ascorbic acid (AA; 10, 20, and 40 mg L^−1^), phloroglucinol (FLO; 50, 100, and 200 mg L^−1^), and polyvinylpyrrolidone (PVP; 100, 200, 400, and 600 mg L^−1^). After 60 days, sprouting, cutting survival, callus formation, rooting, and the number of old and new leaves were assessed. Data were subjected to analysis of variance, and means were compared using the Scott–Knott test. Antioxidant treatments did not promote rhizogenesis in *C. adamantium* cuttings. Nevertheless, the season influenced sprouting, survival, and callus formation, especially during summer and autumn. These results indicate that antioxidant application alone is insufficient to overcome rooting recalcitrance in this species, although it may affect early developmental responses of the cuttings under specific seasonal conditions.

## 1. Introduction

Gabiroba (*Campomanesia adamantium* (Cambess.) O. Berg) is a fruit tree native to the Brazilian Cerrado and belongs to the Myrtaceae family. Its fruits are consumed fresh and are also processed into sweets, jams, juices, and liqueurs, which gives the species economic potential for regional agroindustry and the diversification of native fruit production systems [1]. In addition to its nutritional value, *C. adamantium* has attracted attention because its fruits and other plant parts contain bioactive compounds associated with antioxidant, anti-inflammatory, and other phytotherapeutic properties [2,3,4]. Recent studies also report that cultivation practices can influence production and antioxidant activity in *C. adamantium*, supporting the relevance of studies that connect propagation, plant quality, and oxidative metabolism [5].

The species therefore has relevance not only for fruit production, but also for biodiversity conservation, sustainable use of Cerrado genetic resources, and the development of value-added products based on native plants. Moreover, the utilization of plant resources with medicinal properties supports the development of phytotherapeutic products, promoting human health and aligning with the Sustainable Development Goals (SDGs) established by the United Nations [6].

Currently, the propagation of gabiroba is predominantly carried out through sexual reproduction. However, its seeds are classified as recalcitrant, exhibiting high sensitivity to water loss, which makes long-term storage unfeasible and limits commercial seedling production [7,8]. Furthermore, seed propagation may result in genetically non-uniform plants due to intrinsic genetic variability, compromising seedling quality and plantation productivity [9]. Because clonal propagation has been investigated directly for *C. adamantium* cuttings treated with plant regulators, this constraint also justifies evaluating vegetative alternatives for the species [10]. These limitations highlight the need for studies focused on alternative propagation methods for species of the genus *Campomanesia.*

Asexual propagation offers advantages over sexual propagation by allowing the preservation of the genetic traits of mother plants, ensuring greater uniformity and quality of the propagated material [11]. Among vegetative propagation techniques, stem cuttings are widely used for species with reproductive constraints, including native woody species, but their success varies markedly according to genotype, collection season, cutting type, substrate, mother-plant condition, and the interaction between growth regulators and the propagation environment [12,13,14].

This conditional response is not restricted to *Campomanesia*. Evidence from *Lagerstroemia indica*, *hibiscus*, *blueberry*, *Melaleuca alternifolia*, and other woody species indicates that responses to IBA, substrate, collection season, temperature management, and plant regulators are species- and condition-dependent rather than universally predictable [14,15,16,17].

Many native and woody fruit species therefore exhibit low rooting capacity, which restricts their domestication and commercial exploitation [11,18]. In this context, previous studies with *Campomanesia adamantium* have reported low or variable rooting responses, indicating that this species is difficult to root and that sprouting or cutting survival does not necessarily result in viable rooted plantlets [10,19]. The recalcitrance of *C. adamantium* cuttings to adventitious rooting may be associated with several physiological and anatomical factors, including the physiological condition of the mother plant, tissue lignification, carbohydrate reserves, hormonal balance, endogenous auxin availability, phenolic oxidation, and oxidative responses induced by wounding. These factors may affect tissue viability, cell reprogramming, root primordium initiation, and the establishment of vascular connections required for adventitious root formation [12,20]. Similar limitations have been reported for *Campomanesia phaea*, in which anatomical and histochemical features, including phenolic compounds and sclerenchyma tissues, were associated with reduced adventitious rooting [20].

For *C. adamantium*, studies addressing vegetative propagation remain limited. Available research indicates that the main constraint to seedling production through cuttings is the low rooting potential. Martins et al. [10] evaluated the effects of herbaceous and woody cuttings treated with different concentrations of indolebutyric acid (IBA) (0, 1000, and 2000 mg L^−1^) during December, February, and May, observing a high percentage of live woody cuttings in May. Similarly, Pereira et al. [19] tested IBA concentrations of 0, 800, 1600, and 3200 mg L^−1^ in cuttings of *C. adamantium* and *C. pubescens* during autumn and reported that the regulator did not induce rooting, promoting only shoot emergence.

One factor potentially associated with the low rooting efficiency of gabiroba cuttings is the release of phenolic compounds following tissue sectioning. These compounds may undergo oxidation, leading to tissue darkening and reduced cutting viability [21,22]. In this regard, the application of antioxidants may represent an alternative strategy to mitigate oxidative damage and preserve tissue viability during the initial stages of cutting establishment.

Among the antioxidants commonly evaluated in plant propagation studies, ascorbic acid (AA), phloroglucinol (FLO), and polyvinylpyrrolidone (PVP) stand out for their ability to reduce phenolic oxidation and preserve cellular integrity. These compounds have been used in plant propagation systems because they may reduce phenolic oxidation and tissue browning after wounding, thereby helping to maintain cellular viability during the early stages of cutting establishment [21,23,24]. Previous studies suggest that these compounds can positively influence cutting survival and rooting, although their effects vary according to species, genotype, and environmental conditions [22,25]. However, reactive oxygen species are not exclusively damaging molecules. They can also act as signaling molecules during wound responses, auxin-mediated cell reprogramming, and adventitious root initiation. Therefore, antioxidant application may reduce harmful oxidation, but excessive or poorly timed antioxidant activity may also interfere with oxidative signals required for rhizogenesis [26,27].

The concentrations of ascorbic acid (10–40 mg L^−1^), phloroglucinol (50–200 mg L^−1^), and polyvinylpyrrolidone (100–600 mg L^−1^) were tested as exploratory antioxidant ranges, considering previous reports describing the use of these compounds to reduce browning, modulate oxidative stress, or improve rooting responses in plant propagation systems [21,23,25]. Considering the recalcitrant behavior of *C. adamantium* cuttings and the possible involvement of phenolic oxidation in tissue decline, this study aimed to evaluate the effects of ascorbic acid, phloroglucinol, and polyvinylpyrrolidone on sprouting, survival, callus formation, and attempted adventitious rooting of stem cuttings collected during spring, summer, autumn, and winter. The study was therefore designed to test whether antioxidant treatments could improve developmental responses associated with cutting viability and rooting attempts, rather than to present a successful vegetative propagation protocol.

## 2. Results

During the spring season, the F-test indicated no significant differences among treatments for any of the evaluated variables (Table 1). These results show that the antioxidant treatments, at the tested concentrations, did not influence sprouting, survival, callus formation, rooting, or leaf retention in *C. adamantium* cuttings during this season. Mean rooting was extremely low in spring (0.06 rooted cuttings per plot), reinforcing that antioxidant application did not overcome the rooting recalcitrance of the species.

During summer season, the F-test revealed significant differences only for cuttings with buds (*p* < 0.01) and cuttings with callus formation (*p* < 0.05). For cuttings with buds, treatments T2 (AA 10 mg L^−1^) and T8 to T11 (PVP 100 to 600 mg L^−1^) exhibited the highest mean groupings according to the Scott–Knott test. Regarding callus formation, treatments T2 (AA 10 mg L^−1^), T5 to T7 (FLO 50 to 200 mg L^−1^), and T9 to T11 (PVP 200 to 600 mg L^−1^) showed superior performance, as presented in Table 2. However, these effects on sprouting and callogenesis were not accompanied by a significant increase in rooting. Mean rooting in summer was low (0.42 rooted cuttings per plot) and showed a high coefficient of variation (174.09%), indicating an unstable and biologically limited response.

During the autumn season, significant differences were observed for most evaluated variables, except for the number of new leaves (NFN). For cuttings with buds (EB) (*p* < 0.05), the control treatment, T3 (AA 20 mg L^−1^), and T11 (PVP 600 mg L^−1^) presented the highest mean groupings according to the Scott–Knott test (Table 3). For live cuttings (EV) (*p* < 0.01), treatments T3 and T4 (AA 20 and 40 mg L^−1^), T6 and T7 (FLO 100 and 200 mg L^−1^), and T9 to T11 (PVP 200 to 600 mg L^−1^) differed significantly from the control, exhibiting higher mean values. Similarly, for cuttings with callus formation (EC) (*p* < 0.05), treatments T3 and T4 (AA 20 and 40 mg L^−1^), T6 and T7 (FLO 100 and 200 mg L^−1^), and T9 to T11 (PVP 200 to 600 mg L^−1^) significantly enhanced callus development. For rooted cuttings (EE) (*p* < 0.05), the control, T5 (FLO 50 mg L^−1^), and T8 (PVP 100 mg L^−1^) formed the highest mean group, but the absolute values remained very low, confirming that the treatments did not promote effective adventitious rooting. Concerning the number of old leaves (NFV) (*p* < 0.05), only treatments T5 (FLO 50 mg L^−1^) and T8 (PVP 100 mg L^−1^) exhibited lower mean values compared with the remaining treatments.

In the winter season, the F-test indicated a significant difference only for the variable cuttings with buds (EB) (*p* < 0.01). As shown in Table 2, the control treatment exhibited the highest mean grouping according to the Scott–Knott test, differing significantly from the other treatments. Importantly, both EC and EE were 0.00 for all treatments in winter (Table 1), showing complete absence of callus formation and adventitious rooting during this season under the experimental conditions evaluated.

**Figure 1:**
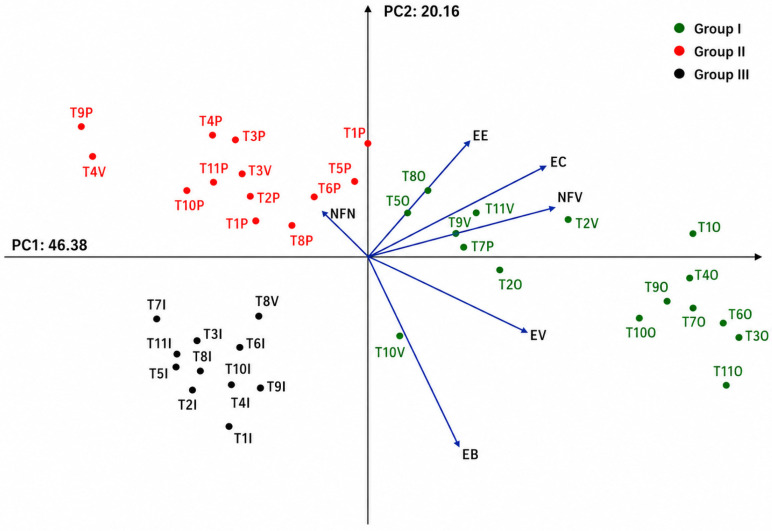
Principal component analysis biplot for cuttings with buds (EB), live cuttings (EV), cuttings with callus formation (EC), rooted cuttings (EE), number of old leaves (NFV), and number of new leaves (NFN) in *Campomanesia adamantium* cuttings collected during spring (P), summer (V), autumn (O), and winter (I). Treatments included the control (T1), ascorbic acid (T2–T4), phloroglucinol (T5–T7), and polyvinylpyrrolidone (T8–T11). The labels Group I, Group II, and Group III are retained only as visual descriptors of ordination tendencies and should not be interpreted as statistically confirmed clusters.

Principal component analysis (PCA) was used as a complementary exploratory analysis. The first two principal components explained 46.38% and 20.16% of the total variance, respectively, for a cumulative variance of 66.54%. Because this cumulative value is below the commonly desirable range of approximately 70–90% for strong dimensional representation, the PCA was interpreted with caution and used only to identify general response tendencies rather than definitive treatment groups. In the biplot, the proximity between vectors indicates a positive correlation among variables, whereas opposite orientations suggest negative correlations. Additionally, variables represented by longer vectors contributed more strongly to the variability explained by the principal components (Figure 1).

The PCA indicated exploratory response tendencies rather than robust treatment groupings. The region labeled Group I in the biplot was associated mainly with autumn treatments and variables related to cutting viability and initial development, such as EB, EV, and EC. This tendency suggests greater maintenance of tissue activity in autumn, but it does not indicate propagation success because rooting remained low. The region labeled Group III was composed predominantly of winter treatments positioned away from EC and EE, consistent with the absence of callus formation and adventitious rooting in winter. The region labeled Group II included spring treatments and some summer treatments and represented an intermediate or weak response. These patterns should be interpreted cautiously because the first two components explained only 66.54% of total variation.

## 3. Discussion

The present study showed that antioxidant treatments modified early developmental responses of *C. adamantium* cuttings, mainly sprouting, survival, and callus formation, but did not promote effective adventitious rooting. This distinction is essential because sprouting and callogenesis indicate temporary tissue viability, but they do not necessarily produce rooted plantlets suitable for establishment after transplanting [12,28,29,30].

Therefore, the main outcome is the persistence of strong rooting recalcitrance under the tested concentrations, immersion period, and seasonal conditions. This response agrees with previous reports for *C. adamantium*, related *Campomanesia* species, and broader woody-plant propagation systems, in which survival, callus formation, or treatment exposure did not necessarily translate into efficient root formation [10,19,20,31,32,33].

The seasonal pattern reinforces the importance of the physiological condition of the mother plant at collection. Antioxidant effects on sprouting were inconsistent: some AA and PVP treatments increased EB in summer and several antioxidant treatments favored EB or survival in autumn, whereas the control showed the highest sprouting response in winter. This variation suggests that antioxidant application did not override seasonal changes in carbohydrate reserves, endogenous hormones, water status, phenolic metabolism, tissue lignification, or cambial activity, all of which can affect cutting viability and rhizogenic competence [12,29,34,35]. Comparable seasonal and substrate-dependent responses have also been reported in *Jatropha curcas* cuttings, reinforcing that propagation environment can condition the expression of rooting competence in woody or semi-woody species [36]. The result is consistent with propagation studies in woody and ornamental species showing that collection period, cutting size, branch source, and environmental conditions can alter the response to growth regulators or other treatments [15,16,36]. Thus, positive responses in EB, EV, or EC should be interpreted as partial effects on cutting maintenance rather than as evidence of propagation success.

Callus formation was stimulated by some treatments, especially in summer and autumn, but it was not accompanied by satisfactory rooting. Although callogenesis is a common wound response and may precede adventitious root formation, it is not equivalent to rhizogenesis [30]. Adventitious rooting requires coordinated cell reprogramming, root primordium initiation, vascular connection with the cutting, and root emergence [12,28,29,35]. In this study, basal calli apparently did not develop into functional adventitious roots, supporting observations that callus formation or sprouting in *Campomanesia* may occur without efficient rooting [19,20,31,32,33].

The low rooting response may reflect combined physiological and anatomical constraints. Wounding can promote phenolic release and oxidation, causing tissue darkening and reduced cellular viability [20,22]. In *Campomanesia phaea*, phenolic compounds and sclerenchyma tissues have been associated with reduced rooting potential [20], and similar barriers may contribute to the recalcitrance of *C. adamantium.* Antioxidants such as AA, FLO, and PVP can reduce oxidative damage and browning [21,22,23,24,25], but the present results indicate that mitigating oxidation alone was insufficient to induce rhizogenesis.

This interpretation is reinforced by the dual role of reactive oxygen species. ROS and reactive nitrogen species are not only damaging molecules; they also act as signals during wound responses, auxin signaling, cell reprogramming, and root primordium initiation [26,27]. Therefore, antioxidants may have preserved tissue viability in some seasons without establishing the hormonal and redox balance required for root induction [12,26,27,28,29,35,37]. The concentrations or immersion time used here may also have been insufficient to affect the critical cellular events involved in adventitious rooting.

Auxin-related explanations should be treated cautiously because no exogenous auxin was applied. Consequently, the absence of rooting cannot be attributed to inhibitory effects of excessive applied auxin. Even so, the results indicate that antioxidant treatment alone did not replace the inductive role usually expected from auxin-based protocols, since auxins interact with cytokinins, ethylene, gibberellins, and other regulators during plant development and root induction [37,38].

Future studies should therefore test antioxidants combined with IBA or NAA, broader concentration ranges, longer or repeated exposure periods, and stricter environmental control. This recommendation is supported by studies in *C. adamantium* and related *Campomanesia* species, as well as by reports in *Lagerstroemia indica*, *avocado*, *hibiscus*, *blueberry*, *Melaleuca alternifolia*, *Eucalyptus*, and other woody systems showing that auxin responses depend on season, cutting type, substrate, light conditions, and complementary treatments rather than on auxin concentration alone [10,14,15,16,17,19,28,31,32,33,34,37,38,39].

The high coefficients of variation, especially for EE and NFN, mainly reflected the low frequency and high dispersion of rooting and new-leaf formation, as previously reported for irregular or low rooting responses in *C. adamantium* and related *Campomanesia* species [10,19,31,32,33]. This pattern is expected when many observations are zero or near zero and supports a conservative interpretation. Future experiments should consider larger sample sizes and statistical approaches suitable for sparse biological responses.

The environmental information available for the greenhouse indicates that temperature varied among seasons in the substrate, greenhouse environment, and leaf surface (Table 4), with higher mean values during spring and summer than during autumn and winter. External relative humidity was obtained from INMET records for regional environmental characterization. The cuttings were maintained under automated irrigation, 60% shading, the BioPlant^®^ commercial substrate, and foliar fertilization with Niphokam^®^ 15 days after experiment establishment. However, relative humidity was not continuously monitored inside the greenhouse, and radiation and vapor pressure deficit were not continuously recorded during cutting establishment. Therefore, temperature can be considered in the interpretation of seasonal responses, but the absence of continuous greenhouse records for humidity, radiation, and vapor pressure deficit still limits a more detailed explanation of cutting dehydration, oxidative stress, and rooting competence [12,28,29,34,35]. In addition, because mother plants were maintained under orchard conditions, spatial heterogeneity in field environments may have contributed to variation in the physiological status of collected branches, an aspect that deserves control or monitoring in future propagation trials [40].

**Table 4:** Temperature values (°C) recorded in the substrate, greenhouse environment, and leaves of *Campomanesia* adamantium cuttings during the experimental periods conducted in different seasons in Jataí, Goiás, Brazil.

Season	Statistical Parameter	Substrate (°C)	Greenhouse Environment (°C)	Leaves (°C)
Autumn	Maximum	32.35	31.58	29.15
	Minimum	11.40	17.43	16.28
	Mean	21.88	24.51	22.72
	Standard deviation	4.68	4.95	4.77
Winter	Maximum	35.48	40.70	35.70
	Minimum	13.00	14.65	9.83
	Mean	24.24	27.68	22.77
	Standard deviation	4.92	5.26	4.77
Spring	Maximum	38.04	41.78	39.52
	Minimum	18.06	17.40	18.30
	Mean	28.05	29.59	28.91
	Standard deviation	5.30	5.44	5.38
Summer	Maximum	42.60	40.30	39.85
	Minimum	16.98	14.84	15.70
	Mean	28.08	28.68	27.84
	Standard deviation	4.63	4.45	4.21

The PCA complemented the univariate analyses by suggesting that autumn cuttings were associated with greater survival and callus formation, whereas winter cuttings showed lower developmental potential, consistent with the absence of callus and rooting in that season. However, because the first two components explained 66.54% of total variance, the biplot should be interpreted only as support for general seasonal tendencies, not as definitive evidence of biological groupings [41,42].

Overall, *C. adamantium* remained highly recalcitrant to adventitious rooting by stem cuttings. Antioxidants improved some viability indicators, but these responses have limited practical value unless they culminate in rooted seedlings. Further work should integrate anatomical, biochemical, hormonal, and environmental evaluations to clarify the mechanisms limiting rooting competence in this species [12,20,26,27,28,29,35,37].

Morphoanatomical approaches, such as those used to describe intra-specific diversity in *Campomanesia pubescens*, could help determine whether callus formation in *C. adamantium* reflects true root primordium initiation or merely wound proliferation without vascular continuity [43]. If conventional cuttings remain ineffective, micropropagation protocols should also be considered as an alternative route, particularly because browning control and successful propagation of recalcitrant woody species often require controlled in vitro conditions rather than isolated exogenous treatments [24].

## 4. Materials and Methods

The experiment was conducted in a greenhouse at the Federal University of Jataí (UFJ), Goiás State, Brazil, located at 17°55′25″ S and 51°42′51″ W, at an altitude of 696 m. According to Köppen’s climate classification, the regional climate is classified as Aw, tropical savanna, and mesothermic, with a rainy season during summer and a dry season in winter.

Vegetative material was obtained from branches collected from mother plants of *Campomanesia adamantium* grown in the UFJ experimental orchard and propagated by seeds. Branches were manually collected using pruning shears and immediately placed in plastic containers filled with a shallow layer of water to prevent dehydration during transport.

A completely randomized design was used, with 11 treatments and three replications. Each replication consisted of five cuttings, totaling 15 cuttings per treatment and 165 cuttings per season. Treatments consisted of three antioxidant compounds at different concentrations plus a control treatment (Table 4).

The antioxidant solutions were prepared in distilled water immediately before treatment application. Cuttings were standardized to 7 cm in length. A bevel cut was made at the basal end to increase the contact area between the vascular cambium and the antioxidant solutions. At the upper end, transverse cuts were performed, maintaining two leaves with the leaf blades reduced to half their original size in order to minimize transpiration.

Treatments were applied by immersing the basal portion of the cuttings in the respective solutions for 15 s. In the control treatment, cuttings were immersed only in distilled water. Subsequently, the cuttings were planted in expanded polystyrene trays (66 × 34 × 6 cm) with 128 cells. The trays were filled with the commercial substrate BioPlant^®^ (Bioplant Misturadora Agrícola, Nova Ponte, MG, Brazil), composed of sphagnum peat, coconut fiber, rice husk, pine bark, and vermiculite, with additives including agricultural gypsum, calcium carbonate, magnesium, and magnesium thermophosphate (Yoorin).

The trays were then placed in a greenhouse covered with a shading screen. Irrigation was provided by an automated inverted micro-sprinkler system, operating three times a day (07:00, 12:00, and 17:00 h) for 1 min per irrigation event. During the 60-day experimental period, cuttings were maintained under the described greenhouse conditions, with automated irrigation and daily monitoring. Fifteen days after the establishment of the experiment in each season, foliar fertilization was performed using 5% Niphokam^®^ foliar fertilizer (10-08-08), at a rate of 25 mL diluted in 5 L of water. The greenhouse was monitored daily throughout the experimental period. Temperature records for the substrate, greenhouse environment, and leaf surface were obtained using an infrared thermometer (InfraRed Thermometer ICEL TD-961, ICEL, Manaus, Amazonas, Brazil) and summarized by season (Table 5). The greenhouse was covered with a shading screen providing 60% light interception. In addition, external air temperature and relative humidity data were obtained from the INMET automatic weather station A016 in Jataí, Goiás, Brazil, to complement the characterization of seasonal environmental conditions. Based on the available hourly records, mean air temperature and relative humidity were 23.70 °C and 77.76% in spring, 23.52 °C and 80.19% in summer, 22.61 °C and 75.83% in autumn, and 25.27 °C and 44.91% in winter, respectively. However, radiation and vapor pressure deficit were not continuously recorded inside the greenhouse, which should be considered when interpreting seasonal effects on cutting dehydration, oxidative stress, and rooting competence.

Values were obtained with an infrared thermometer (InfraRed Thermometer ICEL TD-961). The term “greenhouse environment” refers to the temperature recorded inside the greenhouse during experimental monitoring. Standard deviation was calculated from the temperature records obtained throughout each seasonal experimental period.

Evaluations were performed 60 days after the establishment of the experiment. The following variables were analyzed: cuttings with buds, live cuttings, cuttings with callus formation, rooted cuttings, and the number of old and new leaves. A cutting was considered rooted when at least one visible adventitious root was observed at the cutting base.

Collections and evaluations were carried out in four seasonal periods: spring (18 November 2022–19 January 2023), summer (23 January 2023–27 March 2023), autumn (29 March 2023–30 May 2023), and winter (30 June 2023–31 August 2023).

Data were subjected to analysis of variance (ANOVA) using AgroEstat software version 1.1. When significant differences were detected by the F test, means were grouped using the Scott–Knott test at a 5% probability level. As a complementary analysis, principal component analysis (PCA) was performed to reduce data dimensionality and identify association patterns among the evaluated treatments.

## 5. Conclusions

The antioxidants ascorbic acid (AA), phloroglucinol (FLO), and polyvinylpyrrolidone (PVP) did not improve adventitious rooting of *Campomanesia adamantium* cuttings under the experimental conditions evaluated. However, the season of collection influenced sprouting, survival, and callus formation. Antioxidant treatments affected some early developmental responses, especially during summer and autumn, but these effects did not result in satisfactory rooted plantlets.

The high coefficients of variation observed for some variables may be associated with the genetic variability of the mother plants, a common characteristic of native species that are not yet domesticated and exhibit limited rooting capacity. Overall, although antioxidant treatments contributed to maintaining cutting viability and supported early developmental processes, they were insufficient to induce rhizogenesis in *C. adamantium* under the evaluated conditions. These findings reinforce the complexity of vegetative propagation in *C. adamantium.* Future studies should evaluate broader concentration ranges, different application methods and immersion periods, controlled greenhouse conditions, anatomical and biochemical markers of rooting competence, and the combination of antioxidants with auxins such as IBA or NAA to overcome the rooting recalcitrance of this species.

## Figures and Tables

**Table 1:** Summary of means and analysis of variance for *C. adamantium* (Cambess.) O. Berg propagated by cuttings during four seasons and treated with ascorbic acid (AA), phloroglucinol (FLO), and polyvinylpyrrolidone (PVP).

Season	Statistic	EB	EV	EC	EE	NFV	NFN
Spring	Average values	7.22	2.03	2.03	0.06	2.76	0.46
	F	0.55 ^ns^	0.75 ^ns^	1.33 ^ns^	0.90 ^ns^	0.60 ^ns^	0.86 ^ns^
	*p*	0.8390	0.6689	0.2744	0.5489	0.7970	0.5797
	CV (%)	80.05	59.40	58.03	56.20	70.58	39.17
Summer	Average values	8.73	2.64	2.21	0.42	2.82	0.03
	F	3.75 **	1.82 ^ns^	3.25 *	1.11 ^ns^	1.99 ^ns^	1.00 ^ns^
	*p*	0.0050	0.1170	0.0100	0.3970	0.0870	0.4730
	CV (%)	57.19	33.67	43.10	174.09	58.27	574.46
Autumn	Average values	18.09	3.76	3.64	0.33	5.45	0.09
	F	3.16 *	3.31 **	2.69 *	2.93 *	3.20 *	0.92 ^ns^
	*p*	0.0120	0.0090	0.0250	0.0290	0.0110	0.5330
	CV (%)	23.33	19.66	21.41	127.92	30.78	428.17
Winter	Average values	17.42	2.48	0.00	0.00	0.67	0.18
	F	4.89 **	0.36 ^ns^	-	-	0.75 ^ns^	0.74 ^ns^
	*p*	0.0010	0.9530	-	-	0.6690	0.6810
	CV (%)	14.19	58.61	-	-	154.48	302.77

^ns^ Not significant by the F-test; * and **: significant at 5% and 1% probability, respectively. CV: coefficient of variation. EB: cuttings with buds; EV: live cuttings; EC: cuttings with callus; EE: rooted cuttings; NFV: number of old leaves; NFN: number of new leaves.

**Table 2:** Mean values for variables that differed among treatments in summer and winter in *C. adamantium* propagated by cuttings during the summer and winter seasons and treated with ascorbic acid (AA), phloroglucinol (FLO), and polyvinylpyrrolidone (PVP).

Treatments	Summer EB	Summer EC	Winter EB
T1—Control	5.00 b	1.33 b	24.67 a
T2—AA 10 mg L^−1^	15.33 a	2.33 a	18.33 c
T3—AA 20 mg L^−1^	5.67 b	1.67 b	13.00 c
T4—AA 40 mg L^−1^	2.33 b	0.67 b	16.33 c
T5—FLO 50 mg L^−1^	4.33 b	3.00 a	17.33 c
T6—FLO 100 mg L^−1^	6.33 b	3.33 a	15.33 c
T7—FLO 200 mg L^−1^	1.33 b	3.33 a	15.00 c
T8—PVP 100 mg L^−1^	11.67 a	0.67 b	20.33 b
T9—PVP 200 mg L^−1^	12.67 a	3.00 a	17.00 c
T10—PVP 400 mg L^−1^	17.00 a	2.67 a	19.00 b
T11—PVP 600 mg L^−1^	14.33 a	2.33 a	15.33 c

Means followed by different letters in the columns differ from each other by the Scott–Knott mean grouping at 5% probability. EB: cuttings with buds; EC: cuttings with callus.

**Table 3:** Mean values of *Campomanesia adamantium* cuttings during the autumn season and treated with ascorbic acid (AA), phloroglucinol (FLO), and polyvinylpyrrolidone (PVP).

Treatments	EB	EV	EC	EE	NFV
T1—Control	24.33 a	3.00 b	3.33 b	1.33 a	5.33 a
T2—AA 10 mg L^−1^	15.67 b	3.33 b	3.00 b	0.00 b	5.33 a
T3—AA 20 mg L^−1^	23.67 a	4.00 a	4.33 a	0.33 b	7.00 a
T4—AA 40 mg L^−1^	17.33 b	4.00 a	4.00 a	0.33 b	6.33 a
T5—FLO 50 mg L^−1^	13.00 b	2.67 b	2.33 b	0.67 a	2.00 b
T6—FLO 100 mg L^−1^	17.33 b	4.67 a	4.67 a	0.00 b	6.33 a
T7—FLO 200 mg L^−1^	14.67 b	5.00 a	3.67 a	0.00 b	6.67 a
T8—PVP 100 mg L^−1^	12.00 b	2.67 b	2.67 b	0.67 a	2.33 b
T9—PVP 200 mg L^−1^	19.00 b	4.00 a	4.00 a	0.33 b	5.33 a
T10—PVP 400 mg L^−1^	18.00 b	3.67 a	3.67 a	0.00 b	6.67 a
T11—PVP 600 mg L^−1^	24.00 a	4.33 a	4.33 a	0.00 b	6.67 a

Means followed by different letters in the columns differ from each other by the Scott–Knott mean grouping at 5% probability. EB: cuttings with buds; EV: live cuttings; EC: cuttings with callus; EE: rooted cuttings; NFV: number of old leaves. NFN was not included because no significant treatment effect was detected and the variable is not shown in this table.

**Table 5:** Treatments and concentrations (mg L^−1^).

Treatments	Concentrations (mg L^−1^)
Control	-
Ascorbic Acid	10/20/40
Phloroglucinol	50/100/200
Polyvinylpyrrolidone	100/200/400/600

## Data Availability

The original contributions presented in this study are included in the article. Further inquiries can be directed to the corresponding author.
